# The Population Development of the Red Mason Bee, *Osmia bicornis* L., for Different Types of Nesting Materials

**DOI:** 10.3390/ani14243600

**Published:** 2024-12-13

**Authors:** Barbara Zajdel, Mikołaj Borański, Kornelia Kucharska, Jakub Gąbka

**Affiliations:** 1 Apiculture Division, Institute of Animal Sciences, Warsaw University of Life Sciences–SGGW, 166 Nowoursynowska St, 02-787 Warsaw, Poland; jakub_gabka@sggw.edu.pl; 2 Apicultural Division in Pulawy, The National Institute of Horticultural Research, Konstytucji 3 Maja 1/3, 96-100 Skierniewice, Poland; mikolaj.boranski@inhort.pl; 3 Department of Animal Environment Biology, Institute of Animal Sciences, Warsaw University of Life Sciences–SGGW, 02-787 Warsaw, Poland; kornelia_kucharska@sggw.edu.pl

**Keywords:** *Osmia bicornis*, nest materials, develop population, parasites

## Abstract

*Osmia bicornis* L. is a widespread and valued pollinator species. It is considered to be easy to breed, provided that the nesting material in which the bees build their nests is of sufficient quality and quantity. The aim of this study was to test several different types of nesting materials: reeds and commercial structures, including wood, MDF (Medium Density Fibreboard), plastic, paper or polystyrene. The highest levels of nest cavity occupancy were found in reeds (90%) and grooved MDF (over 80%). We have shown that maintaining mason bee colonies in polystyrene leads to reproductive losses (occupancy only 2% of nesting holes). Mason bees built the most cells in MDF (8.02 cells/hole) and wood (7.34 cells/hole), slightly fewer in plastic (6.83 cells/hole) and reeds (6.74 cells/hole), and the fewest in paper (3.67 cells/hole). The most cocoons per nest were obtained from reed (average 5.47), MDF (4.84) and plastic (4.74). We observed the highest mortality in plastic (2 larvae/hole), and the lowest in reeds (0.92 larvae/hole). In nests made of wood, MDF and paper, large nesting losses were caused by the migration of *Ch*. *osmiae* mites along and through the nest holes. The most hygienic nesting material turned out to be reed and plastic forms.

## Simple Summary

In this study, several different types of nesting materials were tested: common reed and commercial structures made of wood, MDF, plastic, paper, or polystyrene. Red mason bees, *Osmia bicornis* L., most willingly nested in the reed and MDF forms (90% of the holes were occupied). However, they were reluctant to nest in the polystyrene (only 2% of the holes were occupied). MDF, wood, and paper materials turned out to have the lowest level of hygiene, because they flexed and leaked under the influence of moisture, which promoted pollen mold and the migration of *Chaetodactylus osmiae* mites along and through the nest holes. We found the highest mortality in the plastic material (2 larvae/hole) and the lowest in the reed (0.92 larvae/hole). The materials in which the bees’ best reproductive results were obtained were the reed and plastic forms (despite the high level of larve mortality): the authors recommend these two types of materials for the commercial breeding of red mason bees.

## 1. Introduction

The production of fruits, vegetables, and seeds from 87 of the world’s leading food crops is dependent on animal pollination [1]. Honeybees are the main pollinator group in the temperate zone [2]. The intensification of agriculture, use of pesticides, increases in crop area, and landscape homogenization all contribute to a decrease in the diversity of wild pollinators in agricultural areas [3,4,5,6]. This in turn negatively affects plant pollination levels, causing a decrease in fruit and seed production [3,7,8,9]. Honeybee (*Apis mellifera* L.) colonies, each of which contain several thousand foragers, are often used to pollinate crops. Honeybees have long foraging ranges [10] and collect pollen from various sources [11,12]. For several decades, there has been a noticeable decline in honeybee colonies observed worldwide [13]. The cause is not clear, but the most frequently cited reasons are pests and diseases, bee management and breeding, climate change, and the pesticides used in agriculture [14]. This has led to the search for alternative pollination methods using other bee species, including those belonging to the *Osmia* genus from the Megachilidae family [15,16,17]. A major advantage of bees from the *Osmia* genus is their short flight distance from the nest, about 600 m [18,19], and often only 50–300 m during the period when plants flower intensively [17,20]. Species of the *Osmia* genus are generalists, pollinating over 150 plant species [21,22], thus positively influencing the quantity and quality of crops. This is why they have been successfully used for many years to pollinate various crops, such as orchards [23,24,25,26,27,28,29,30,31], rapeseed [32], blackcurrant [23], and strawberries [33]. Recent reports show that mason bees are also used to pollinate forest orchards of European seed trees—*Tilia cordata* Mill. and *Prunus avium* L.—increasing their seed yield [34]. Vicens and Bosch (2000) [35] showed that *Osmia* bees actively forage at lower temperatures (from 10 °C) than honeybees (*Apis mellifera*) (from 12 °C) and also fly under strong wind and light rain. The pollination efficiency of bees of the *Osmia* genus for some plants, e.g., pears and almond, is higher than that of A. mellifera [36,37]. Additionaly *O. bicornis* is its lower sensitivity to some insecticides compared to the honeybee [38].

*Osmia* bees is univoltine species, and their activity is observed during the period of the greatest demand for pollinators (from early spring to early summer) [21]. Recent studies have shown that the use of methoprene (an analogue of juvenile hormone) allows controlling the diapause period of the mason bee, which allows the activation of adult bees at different times [39,40]. The ability to control the diapause period will allow the use of *O. bicornis* for pollination of crops in greenhouses in autumn, winter and early spring.

Bees of the *Osmia* genus show great plasticity in choosing nesting sites. Most representatives of this genus nest in cavities in dead wood or plant stems [41]. In addition, some species nest in empty snail shells [42,43] or build ground nests (surface or subterranean) [44], or use nests built by other insects (including *Anthophora* bees and *Sceliphron* wasps) [41]. Some representative species of this genus are synanthropic and have been nesting in the cracks of rural buildings for years [21].

One of the most important conditions for breeding mason bees is providing the bees with appropriate nesting material. In Poland, the most popular species of wild bees used for pollinating crops is *Osmia bicornis*. These bees build cavity-nests using holes in various substrates [21,44,45]. They are eager to inhabit reed tubes and drilled wooden blocks [21]. According to Michołap et al. (2020) [46], the optimal length for nest holes is between 15 cm and 20 cm. The bees most often choose holes with diameters of 5–7 mm [40] or 5–8 mm [23,47]. The diameter and length of the holes play an important role in regulating the sex ratio of the population [48,49]. The holes should not be shorter than 15 cm, because this increases the number of males in the bee population [49].

Currently, the most commonly used nesting materials in mason bee breeding are cut, empty plant stems, mainly the common reed (*Phragmites australis* L.) or bamboo (*Bambusa* Shreb.). However, Madras-Majewska et al. (2011) [50] showed that reed nests used in mason bee breeding cannot be used for more than one season due to the enormous damage caused by parasitic nest fauna. The fauna that inhabits mason bee nests is an inseparable element of breeding these bees. The number of faunal species and the intensity of their occupation of the nesting chambers increases in proportion to the number of years the bees are kept in a given habitat [28,51]; this also depends on the type of habitat itself [52,53,54]. Nesting materials tested by Wilkaniec and Giejdasz (2003) [45] were mostly disposable (paper and plastic tubes, plastic insulation); only blocks of wood and cork could be reused. Preparing disposable nests is quite laborious and time-consuming; therefore, the search is still ongoing for nesting materials that can be used for breeding over several years, provided that good bee reproduction parameters and health are maintained. In recent years, proposals for new nesting materials for solitary bees have appeared on the Polish/European market, mainly in formats that are arranged in stacks to create nest holes.

The aim of this study was to determine the willingness of bees to nest in reusable structures made of different types of nesting materials, as well as to assess the reproductive performance of the bees, and the materials’ hygiene and suitability for multiple reuse. We compared the most popular commercially available (on the European market) reusable nesting materials (made of plates arranged in stacks and creating nesting holes) for cavity-nesting solitary bees—made of MDF (Medium Density Fibreboard, wood-based product), wood, plastic, paper, and polystyrene—with natural reed tubes, commonly used for *Osmia* breeding.

## 2. Material and Methods

### 2.1. Study Area

**Figure 1:**
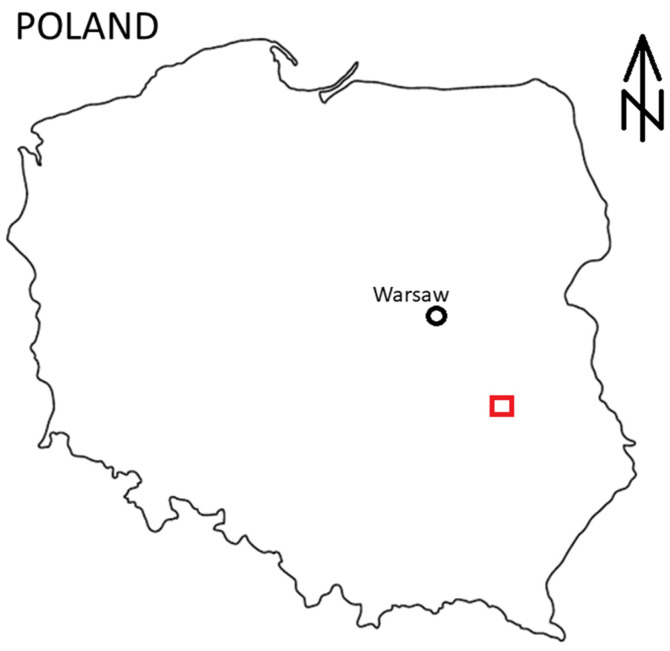
The red box indicates the location of the study site (GPS coordinates 51°40′94″, 21°98′15″).

This study was conducted in an experimental apiary belonging to the Apicultural Division, The National Institute of Horticultural Research in Puławy, located in the south-eastern part of Poland (Puławy County 51°40′92″, 21°98′12″, Figure 1) during 2016–2018.

### 2.2. Material

The research material consisted of cocoons of the red mason bee *Osmia bicornis*, which had been bred at the National Institute of Horticultural Research of Apiculture Division in Puławy.

**Figure 2:**
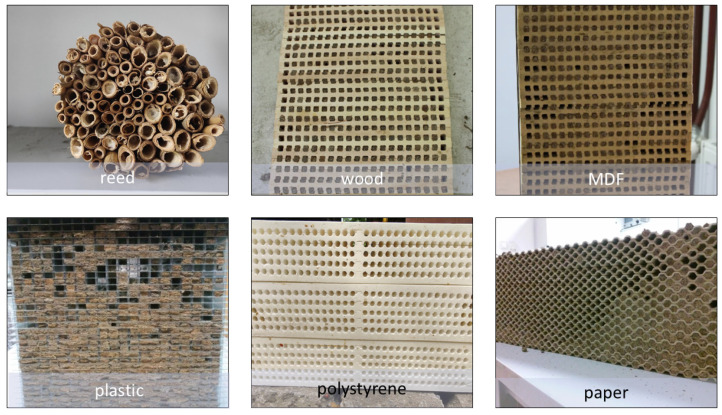
Types of nesting materials used in the experimental.

The experiment used six types of nesting materials for the mason bees:

- Reed tubes—a natural material with a hole diameter of 6–8 mm and tube length of 15 cm (control group).
- Commercial lime-tree wood boards, free of preservatives, made with 7 mm wide grooves and stacked to create holes 7 × 7 mm in diameter and 15 cm long.
- Commercial MDF boards, free of preservatives, made with 7 mm wide grooves and stacked to create holes that were 7 × 7 mm in diameter and 15 cm long.
- Commercial nests made of plastic plates that were arranged in stacks to create holes 15 cm long, with a rectangular cross-section measuring 6 × 8 mm.
- Commercial polystyrene nests, arranged in stacks to create holes with a diameter of 6 × 7 mm and a length of 15 cm.
- Paper plates made of cellulose pulp, arranged in stacks to create holes with a diameter of 7 × 8 mm and a length of 15 cm. (Figure 2).

Except for the control nests, the panels were layered one on top of the other so that the grooves created nest holes. The panels made of MDF, wood, and paper were pressed tightly together and fastened with clamps so that the tiles formed a compact structure and the holes were tight. The remaining plastic and polystyrene panels had a built-in “click” system, which ensured the correct fit and tightness of the structure.

At the beginning of April, six boxes, each containing one type of nesting material consisting of 700 nest holes, were placed in the apiary. The boxes were spaced about 300 m apart, at a height of 1 m, in places with moderate sunlight. This distance ensured that the bees would build nests in specific nesting materials while maintaining the same nectar, pollen and weather conditions (temperature, wind, cloud cover). The boxes were placed with the outlets facing south-west or south at a height of 1 m, in places with moderate sunlight. Each box contained 1000 mason-bee cocoons. The bees emerged and were active from mid-April to mid-June.

The bees were active from mid-April to mid-June. After the active were completed, the nesting materials remained in the boxes until the end of September and were then taken to the laboratory for analysis. The materials were opened and the following factors were determined [51,54]:

- Nest cavity occupancy (the number of occupied holes);
- Emergence rate (%)—percentage of bees that emerged from 100 randomly cocoons;
- Population growth rate (n) ratio of the number of cocoons collected after the season (progeny generation) to the number of cocoons placed in the site/box in spring (parental generation);
- Nesting chambers;
- Fully formed cocoons;
- Healthy cocoons (i.e., those not infected with the mite *Chaetodactylus osmiae* Dufour);
- Mortality of larvae and pupae—total number of dead larvae and pupae to the number of occupied nest tubes;
- The number of parasitized chambers (*Ch*. *osmiae*, *Cacoxenus indagator* Loew, *Monodontomerus obscurus* Westwood).

The fully formed cocoons that had been removed from the nests were stored in perforated boxes in cold storage at 4 °C until mid-April of the following year. In mid-April of both years of the study, a check was carried out for the number of bees that had been emerged from 100 randomly selected cocoons each type of nesting material (emergence rate).

### 2.3. Statistical Analyses

To assess the attractiveness of nesting material to solitary bees (relationship between the type of nesting material and nest cavity occupancy), we used Pearson’s chi-squared test (using multiple comparisons with Benjamini–Hochberg correction, *p* < 0.05). The potential effect of nesting material on the reproductive parameters of *O. bicornis* was investigated using the Kruskal–Wallis test (with multiple comparisons of the mean rank for all samples—Dunn’s test with Bonferroni correction, *p* < 0.01). The distribution of the analyzed traits differed significantly from the normal distribution, as confirmed by the Shapiro–Wilk test (*p* < 0.05). Calculations were carried out using PQStat Software, v.1.8.6.122.

## 3. Results

**Figure 3:**
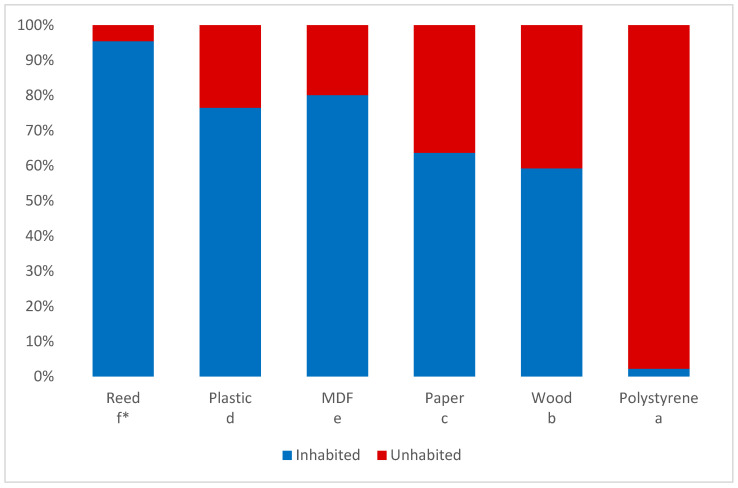
Nest cavity occupancy for different types of material. * Different letters indicate significant differences between groups (Pearson’s chi-squared test *p* < 0.05, df = 5).

The type of nesting material significantly influenced the number of occupied holes (ꭓ^2^ = 4708.91, *p* < 0.01, df = 5). Bees were most likely to build their nests in the reed tubes. The settlement of the other types of material differed significantly from the reed and from each other. The least attractive nesting material for female bees was polystyrene—the bees built their nests in only 2% of the available nesting channels (Figure 3). 

The average emergence rate was high for all nesting boxes and ranged from 94.56% to 96.21%. The three-year population growth rate was highest for the reed tubes. Satisfactory rearing results for *Osmia bicornis* were also obtained for plastic and MDF forms. In the case of polystyrene and paper forms, there was a decrease in the *Osmia bicornis* population (Table 1).

Due to the very low settlement rate for the polystyrene forms, this material was excluded from further analyses as an unattractive material for red mason bees (*O*. *bicornis*). Despite the same nest channel lengths, there were differences in the number of created breeding chambers in the different types of nesting materials (H_(4, 8042)_ 1273.27, *p* < 0.01). The bees built more breeding chambers in nesting material made from MDF. In this respect, the plastic forms did not differ from the reed tubes. The total number of cocoons (H_(4, 8042)_ = 1360.20, *p* < 0.01) and the number of healthy cocoons (H_(4, 8042)_ = 1636.82, *p* < 0.01) were statistically highest in the reed tubes. For all forms, the best results were obtained for plastic. Mortality varied across the different types of nesting materials (H_(4, 8042)_ = 577.54, *p* < 0.01). More larvae/pupae died in the forms made from plastic, while the lowest value for this parameter occurred in reed tubes (Table 2).

The level of nesting-chamber parasitization varied across the different materials (H_(4, 8042)_ = 2479.69, *p* < 0.01). Significantly fewer cells were occupied by parasites in the plastic forms. Also, for the three most relevant parasitoid species—*C*. *indagator* (H_(4, 8042)_ = 526.62, *p* < 0.01), *Ch*. *osmiae* (H_(4, 8042)_ = 1751.21, *p* < 0.01), and *M*. *obscurus* (H _(4, 8042)_ = 70.81, *p* < 0.01)—the plastic forms showed the lowest levels of infested breeding chambers. There were no differences between the reed tubes and the other forms in relation to the number cocoons infected by *M*. *obscurus*. However, for *C*. *indagator* and *Ch*. *osmiae*, forms other than plastic were statistically more infested than the reed tubes. The nests in forms made of wood and wood-based composite materials (MDF) had particularly high infestation of *Ch*. *osmiae* (Table 3).

## 4. Discussion

Next to Apidae, Megachilidae are the most important group of Hymenoptera for pollinating crops [15,16,17].

In recent years, there has been great interest in increasing the population of wild bees by providing additional nesting sources in the form of bee hotels [55,56]. Over the years, various materials, such as glass tubes [57,58,59,60] and holes drilled in wooden blocks [61,62], have been tested for durability, ease of maintenance, and utilization by different species. Many studies have been written on pollinator hotels, focusing on the attractiveness of a given nesting material, the location of the nest relative to cardinal directions, and the species of insects that inhabit them [51,63,64].

In this study, we have shown a more commercial approach to *Osmia bicornis* breeding, because farmers’ interest in using this species for crop pollination is constantly growing. Breeding mason bees in artificial nests is widely considered to be easy and much less expensive when compared to maintaining honeybee colonies. Breeding success and obtaining healthy cocoons every year largely depends on the quality of the nesting material. Until now, the most popular nesting material for mason bees in Poland has been the common reed (*Ph. australis*). Reed has also been used in many scientific studies conducted on mason bees [22,23,52,53], and our results showed that reed was the most popular nesting material for *Osmia bicornis*. The level of reed occupation was over 90% of available nest holes. Gonzales-Zamora et al. (2021) [55] and Khan et al. (2024) [64], in their studies on pollinator hotels, showed that natural bamboo tubes were one of the most attractive nesting materials for wasps. Wilkaniec and Giejdasz (2003) [45] showed that the red mason bees’ occupation rate of reed was 100%.

The second most attractive nesting material was grooved MDF boards (occupancy over 80%). Our research showed that mason bees also willingly nested in plastic nests (occupying about 80% of available holes). Similar results were obtained by Wilkaniec and Giejdasz [45] for plastic drinking straws and heat shrinkable electrical insulation—the levels of occupation were 79.3% and 86%, respectively. These authors obtained acceptance of 100% for plastic print sheet, which indicates that this type of nesting material was highly attractive.

In our study, for paper and wood nests, mason bees occupied about 62% and 58% of available holes, respectively. These results are similar to those obtained by Wilkaniec and Giejdasz [45], where mason bees colonized wooden blocks at a level of 40.6% to 81.3% during their two-year study, while paper tubes were only 36% colonized.

Our results showed that polystyrene plates are not suitable for breeding mason bees, as the bees colonized only 2% of nest holes. We demonstrated that maintaining mason bee colonies in polystyrene leads to breeding losses. Interestingly, similar polystyrene plates have been successfully used in the breeding of other bee of the Megachilidae family—*Megachile rotundata* [65,66].

In the breeding of mason bees, such parameters as the number of chambers built by the female and the number of cocoons obtained from one nest (from one nest hole) are very important. The length of the nest hole has a significant influence on these parameters. It is difficult for us to compare our results with other results from the literature due to the differing lengths of nest holes used in the studies (e.g., Wilkaniec and Giejdasz [45]—22 cm, our studies—15 cm). Of the materials we tested, the mason bees built the most cells in MDF (8.02 cells/hole) and wood (7.34 cells/hole), slightly less in plastic (6.83 cells/hole) and in reed (6.74 cells/hole), and the least in paper (3.67 cells/hole). Wilkaniec and Giedasz [45] showed that mason bees built the most chambers in wood (8.9 cells/hole) and reed (6.9 cells/hole), while in the remaining materials, i.e., plastic and paper tubes, the bees built from 3.5 to 4.1 nesting cells. Bosh [67] showed that the most suitable nesting material for *Osmia cornuta* bees was wood blocks, for which the best reproductive results obtained were comparable to reed and bamboo.

The largest numbers of cocoons per nest hole was obtained for reed (on average 5.47), MDF (4.84), and plastic (4.74), while the least were for wood and paper (3.49 and 1.77, respectively). Wilkaniec and Giejdasz [45] obtained different results from ours, where they showed the largest number of cocoons were in wood and reed (5.8 and 4.3, respectively), and the least were for paper and plastic (2.3 and 0.5, respectively). Wilkaniec and Giejdasz [45] also tested cork forms, for which they obtained 4.7 cocoons per nest hole. Cork is a wood-based material similar to MDF, and the results obtained by the authors (4.7 cocoons/hole) were similar to those we obtained for MDF (4.84 cocoons/hole).

The average production of bees per nest tube offered was greatest in either wood or reed stems. Managing *O*. *bicornis* was easiest in the disassembled nests made of wood (45). In our study, the highest bee production was observed in reed, MDF, and plastic materials. The differences between our findings on the number of cocoons per nest and those reported by Wilkaniec and Giejdasz [45] may be attributed not only to the type of nest material and the hole length (22 cm) but also to the number of nest holes available per cocoon (bee). In Wilkaniec and Giejdasz’s study (2003), the number of cocoons per nest hole ranged from 3.8 to 6.1 [45], whereas in our study, it was lower at 1.4 cocoons per nest hole. The low ratio of nests per cocoon in Wilkaniec and Giejdasz (2003) [45] may have facilitated usurpation of partially constructed nests, potentially contributing to the higher number of cocoons per nest. Although *Osmia bicornis* builds and provisions its own nests, nest usurpation by conspecific females has been documented in other *Osmia* species [17,68].

In the analysis of our results, we included the parameter healthy cocoons, which indicated cocoon losses caused mainly by the nest parasite mite *Chaetodactylus osmiae*. The greatest differences between the total number of cocoons and the number of healthy cocoons were shown for MDF, paper, and wood. These materials turned out to be the least hygienic. Wood and MDF boards come apart under the influence of moisture, which allowed *Ch. osmiae* mites to pass from cell to cell or along and across nest holes. Similar results were obtained by Bosh (1992) [67]. However, results that were completely opposite were obtained for wood by Wilkaniec and Giejdasz (2003) [45], who described grooved boards has giving the highest number of cocoons and the highest percentage of hatchings. The lowest number of healthy cocoons was obtained in nests made of paper (1.29 larvae/tube), which absorbed moisture and caused pollen to become moldy inside the nesting chambers. Pollen that becomes infested with mold is considered to be the main factor in larval mortality [69]. In our results, the highest mortality was recorded for plastic (average 2 larvae/one nest hole). We also observed moldy pollen inside the nesting chambers. Larvae developed best in reed, where the mortality rate was below one larvae/tube.

A very important factor in red mason bees breeding is the hygiene of the nesting material. Even with an abundance of melliferous plants and high utilization of nesting material, breeding success will not be achieved if the material favors the development of parasites. The material that most limited the presence of the three most common nest parasites and kleptoparasites, i.e., *C. indagator*, *Ch. Osmiae*, and *M. obscurus*, turned out to be plastic. The results for plastic were even better than for the control material, reed. For the remaining materials, parasites developed well, causing large losses in the number of cocoons. This was especially true for wood and MDF, where the secondary infection of cocoons by *Ch. osmiae* mites was observed through the migration of mites along and across the nest channels. Mite migration among *Osmia* nests was reported also by Park et al. [70], Joshi et al. [71] proposed a modification of wood nesting material (paper liners in nesting channels) that reduce migration of mites among nests.

Summarizing our results, it can be stated that mason bee populations develop best in reed, and no commercial material can compare to it, as evidenced by it having the highest population growth rate of 3.78. From other publications, in which the mason bee was bred in reed, population growth was similar to the results obtained by Fliszkiewicz et al., 3.17 [53], and Zajdel et al., 3.42 [72], in agricultural areas. Many studies have indicated that a complex of specific environmental conditions has a significant impact on population growth [52,53,54,66].

We believe that mason bee breeders who want large annual population increases should use reed nests. The material that came second in terms of obtaining healthy cocoons in relation to hygiene was plastic forms. These nests can be successfully recommended to farmers and fruit growers, especially since these are structures that can be used for many seasons and can be disinfected. The use of reed involves replacing the material annually with new reed [50] (searching for and trimming reeds or the annual purchase of new reed packages), which generates costs and is more labor-intensive. An additional advantage of plastic forms is the fact that females obtained from these nests have a greater body mass than those obtained from reed nests [73].

Wood and MDF forms were eagerly inhabited by bees, but they were not hygienic and allowed parasitic fauna to develop These materials still require refinement by manufacturers, especially by improving the tightness of the structure to prevent the migration of parasites inside the nests.

## 5. Conclusions

The type of nesting material has a significant impact on the development of the *O. bicornis* L. mason bee population. The choice of nesting material affects the reproductive results of bees (the number of cocoons, larval mortality) and limits the damage caused by parasites. Bees prefer natural materials for building nests, such as reed tubes, MDF boards or wood.

Regardless of the choice of nesting material for bees, breeders should take care of the maximum fit of the formats to ensure the tightness of the holes. Tight nesting holes will limit the migration of the *Chaetodactylus osmiae* mite, which causes significant losses to the mason bee population.

## Figures and Tables

**Table 1:** *Osmia bicornis* rearing rate for the different types of nesting materials.

	Type of Material					
	Reed	Plastic	MDF	Paper	Wood	Polystyrene
Emergence rate (Eg) *	95.26	94.56	96.21	96.14	95.78	95.84
Population growth rate (Pg)	3.78	2.68	1.82	0.78	1.19	0.02

* average Eg for three years of study.

**Table 2:** Number of breeding chambers, cocoons and healthy cocoons, and mortality rate for the different types of nesting materials.

		Type of Material				
		Reed	Plastic	MDF	Paper	Wood
Nesting chambers (*n*)	Mean ± SD	6.74 ± 1.58 ^b^	6.83 ± 3.32 ^b^	8.02 ± 2.95 ^d^	3.67 ± 3.27 ^a^	7.34 ± 3.24 ^c^
	Mdn	7	7	8	3	8
	IQR	2	6	4	5	5
	Min–max	1–12	1–14	2–14	1–14	2–14
Fully formed cocoons (*n*)	Mean ± SD	5.47 ± 2.15 ^d^	4.74 ± 3.49 ^c^	4.84 ± 3.19 ^c^	1.77 ± 2.77 ^a^	3.49 ± 3.32 ^b^
	Mdn	6	5	5	0	3
	IQR	3	5	6	2	6
	Min–max	0–12	0–14	0–13	0–13	0–12
Healthy cocoons (*n*)	Mean ± SD	5.24 ± 2.16 ^d^	4.71 ± 3.48 ^c^	3.05 ± 3.27 ^b^	1.67 ± 2.66 ^a^	2.76 ± 3.17 ^b^
	Mdn	6	5	2	0	2
	IQR	3	5	6	2	5
	Min–max	0–12	0–14	0–12	0–13	0–12
Mortality (*n*)	Mean ± SD	0.92 ± 1.29 ^a^	2.01 ± 1.75 ^d^	1.11 ± 1.46 ^b^	1.29 ± 1.27 ^c^	1.65 ± 1.80 ^c^
	Mdn	0	2	1	1	2
	IQR	1	2	2	2	3
	Min–max	0–8	0–10	0–8	0–9	0–9

Different letters indicate significant differences between groups (Kruskal–Wallis test, *p* < 0.01, df = 4).

**Table 3:** Number of chambers occupied by parasite species (*C*. *indagator*, *Ch*. *osmiae*, *M*. *obscurus*) across different types of nesting materials.

		Type of Material				
		Reed	Plastic	MDF	Paper	Wood
*C*. *indagator*	Mean ± SD	0.16 ± 0.53 ^b^	0.02 ± 0.21 ^a^	0.80 ± 1.66 ^d^	0.33 ± 0.77 ^c^	0.78 ± 1.61 ^d^
	Mdn	0	0	0	0	0
	IQR	0	0	0	0	0
	Min–max	0–6	0–4	0–11	0–8	0–9
*Ch*. *osmiae*	Mean ± SD	0.18 ± 0.56 ^b^	0.07 ± 0.47 ^a^	2.98 ± 3.69 ^e^	0.54 ± 0.99 ^c^	1.99 ± 2.66 ^d^
	Mdn	0	0	0	0	0
	IQR	0	0	6	1	3
	Min–max	0–5	0–7	0–13	0–8	0–14
*M*. *obscurus*	Mean ± SD	0.07 ± 0.31 ^bc^	0.01 ± 0.12 ^a^	0.08 ± 0.46 ^b^	0.12 ± 0.65 ^bc^	0.16 ± 0.70 ^c^
	Mdn	0	0	0	0	0
	IQR	0	0	0	0	0
	Min–max	0–4	0–2	0–6	0–9	0–8
All parasites	Mean ± SD	0.40 ± 0.83 ^b^	0.10 ± 0.53 ^a^	3.86 ± 3.70 ^e^	1.00 ± 1.38 ^c^	2.93 ± 3.01 ^d^
	Mdn	0	0	3	0	2
	IQR	1	0	7	2	5
	Min–max	0–6	0–7	0–14	0–9	0–14

Different letters indicate significant differences between groups (Kruskal–Wallis test, *p* < 0.01, df = 4).

## Data Availability

The data presented in this study are available on request from the corresponding author.
